# Flexural Strength Comparison of Silorane- and Methacrylate-Based Composites with Pre-impregnated Glass Fiber

**Published:** 2016-06

**Authors:** Maryam Doozandeh, Ali Asghar Alavi, Zahra Karimizadeh

**Affiliations:** 1 Dept. of Operative Dentistry, School of Dentistry, Shiraz University of Medical Sciences, Shiraz, Iran.; 2 Dept. of Operative Dentistry and Biomaterials Research Center, School of Dentistry, Shiraz University of Medical Sciences, Shiraz, Iran.; 3 Undergraduate Dental Student, Biomaterials Research Center, School of Dentistry, Shiraz University of Medical Sciences, Shiraz, Iran.

**Keywords:** Flexural Strength, Pre-impregnated Glass Fiber, Nanohybrid Composite, Silorane-based Composite

## Abstract

**Statement of the Problem:**

Sufficient adhesion between silorane/methacrylate-based composites and methacrylate impregnated glass fiber increases the benefits of fibers and enhances the mechanical and clinical performance of both composites.

**Purpose:**

The aim of this study was to evaluate the compatibility of silorane and methacrylate-based composites with pre-impregnated glass fiber by using flexural strength (FS) test.

**Materials and Method:**

A total of 60 bar specimens were prepared in a split mold (25×2×2 mm) in 6 groups (n=10). In groups 1 and 4 (control), silorane-based (Filtek P90) and nanohybrid (Filtek Z350) composites were placed into the mold and photopolymerized with a high-intensity curing unit. In groups 2 and 5, pre-impregnated glass fiber was first placed into the mold and after two minutes of curing, the mold was filled with respective composites. Prior to filling the mold in groups 3 and 6, an intermediate adhesive layer was applied to the glass fiber. The specimens were stored in distilled water for 24 hours and then their flexural strength was measured by 3 point bending test, using universal testing machine at the crosshead speed of 1 mm/min. Two-way ANOVA and post-hoc test were used for analyzing the data (*p*< 0.05).

**Results:**

A significant difference was observed between the groups (*p*< 0.05). The highest FS was registered for combination of Z350 composite, impregnated glass fiber, and application of intermediate adhesive layer .The lowest FS was obtained in Filtek P90 alone. Cohesive failure in composite was the predominant failure in all groups, except group 5 in which adhesive failure between the composite and fiber was exclusively observed.

**Conclusion:**

Significant improvement in FS was achieved for both composites with glass fiber. Additional application of intermediate adhesive layer before composite build up seems to increase FS. Nanohybrid composite showed higher FS than silorane-based composite.

## Introduction

Resin-based composites have been the material of choice for most restorations in dental practice during the last decade.[1] Since the development of composite resin, several modifications have been made to reduce their limitations like mechanical deficiencies, polymerization shrinkage, and degradation in oral environment.[2-3] Impregnated fiber-reinforced composite (FRC) resin can be used as filler into the resin composite matrix, or separately in conjunction with the resin composite. Both forms are acceptable approaches to enhance the mechanical properties of composite resin to function well in oral cavity.[1, 4]Many researches supported the satisfactory handling properties and adequate clinical performance of FRC.[5-6] They could be used in periodontal splint, avulsed teeth splint, endodontic post and cores, orthodontic retainers reinforcement and recently, in fixed partial dentures.[7-8]

Fiber impregnation with adhesive resin may be an important factor in developing the adhesive strength between the fiber and composite materials by transferring the loading forces from the resin matrix to the fiber.[9] The two resin systems which are used for impregnation of fiber employ photopolymerizable dimethacrylate monomer and a combination of dimethacrylate monomer resin along with liner polymer which form the semi-interpenetrating polymer network.[10-11] Fiber impregnation may be performed by the manufacturer or by the clinicians.

In addition to adequate resin impregnation, the compatibility of veneering composite with different fibers is another important part in achieving the ideal mechanical properties of FRC system.[12] Different composite resin systems can be used in conjunction with fiber to enhance the advantages of fibers in increasing the physical and mechanical properties. Tanoue *et al.* declared that particular type of composite material should be selected for optimal fiber-composite combination. They found that the fiber-microhybrid composite demonstrated the highest flexural strength.[12] Another study showed that the fiber and composite type significantly influenced the flexural strength of FRC. In that study, combination of glass fiber and Z250 composite was found to have the highest flexural strength.[13] Eronat *et al.* noticed a significant increase in flexural strength in incorporation of fiber. On the other hand, flexural strength of both hybrid and microfilled composite were enhanced when they were reinforced with glass fiber.[14] The insertion of fibers also significantly reduced the microleakage in class II composite restorations.[15-17]

Recently, ring-opening silorane-based composites have been increasingly used to overcome the shortcoming of methacrylate resin composites and improve their clinical performance.[18]

It was demonstrated that the pre-impregnated glass fiber along with silorane-based composites can create better result in cuspal deflection and fracture resistance of endodontically treated premolar.[19]

Polacek *et al.* showed that application of an intermediate layer of adhesive resin on the pre-impregnated glass fiber before composite build-up yielded higher shear bond strength (SBS).[20] Many researchers postulated that flexural strength is a good indicator for clinical performance of FRC, especially in FRC fixed partial denture.[12-14,21]

To the best of the authors’ knowledge, no information was found on the flexural strength and compatibility of silorane-based composite with methacrylate resin impregnated glass fiber in literature. So the purpose of this study was to compare the flexural strength and failure mode of a silorane-based composite and a nanofilled resin composite per se and in conjunction with monomer pre-impregnated glass fiber as a veneering composite; and also to evaluate the effect of initial application of adhesive resin to monomer impregnated glass fiber before composite veneering. 

## Materials and Method

Two commercially available direct composites were selected for this study; Filtek Z350 (3M; ESPE, USA) as a methacrylate-based nanohybrid composite, and Filtek P90 (3M; ESPE, USA) as a silorane-based composite, both with A3 color from Vita shade guide. A multidirectional monomer pre-impregnated glass fiber (Ribbon; Angelus, Brazil) was also selected.

To determine the flexural strength, a stainless steel mold (2×2×25 mm) was fabricated according to ISO 4049 and used to obtain 60 rectangular composite bars in 6 groups (n=10) as follows.

In groups 1 and 4 (control groups), Filtek Z350 and Filtek P90 composites were placed into the mold in one increment and were completely condensed. The surface of the mold was covered with a Mylar strip, light polymerized for 40 seconds with VIP Junior curing unit (Bisco; Schaumberg, USA) at a 600 mW/cm^2^ light intensity, and cured in three separate overlapping techniques. After 10 minutes, the composite bars were removed from the mold and the above-mentioned curing procedure was repeated on the other side surfaces.

In groups 2 and 5, a 25-mm pre-impregnated glass fiber was cut and put at the bottom of the mold. Several laboratory studies demonstrated that the maximum flexural strength would be acquired when the fiber is placed at the bottom of the specimen.[13] After photopolymerizing the glass fiber for 1 minute (to simulate the clinical application), both composite resins were applied into the mold and light cured just like the control groups by using the same polymerizing device.

In groups 3 and 6, the glass fiber was photopolymerized as it was in group 2 and 5. An intermediate layer of adhesive resin, which was Margin bond (Coltène Whaledent; Germany) in group 3 and Silorane adhesive bond (3M; ESPE, USA) in group 6, has been applied to the glass fiber in one coat and gently air-thinned. Then, the composites were put into the mold similar to previous groups.

The specimens were stored at 37°C for 24 hours and tested on a three point bending device by using a universal testing machine (Instron; Zwick/Roell, Germany) at a crosshead speed of 0.5 mm/min. The fiber-composite specimens were oriented in the jig with the fiber at the bottom. 

The flexural strength was calculated considering the peak load, length, and cross-section of specimen.

**Figure 1:**
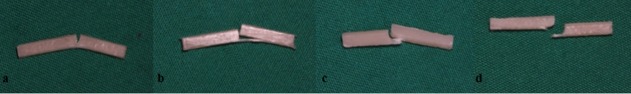
Representation of failure modes. a: cohesive failure in composite. b: adhesive failure in composite-fiber interface. c: partial breakage of the composite and fiber. d: complete breakage of the composite and fiber

For each bar specimens, the loading force was applied until the composite fractured. The failure mode was observed and categorized as cohesive failure in composite (A), adhesive failure in composite-fiber interface (B), partial breakage of the composite and fiber (C), and complete breakage of the composite and fiber (D). (Figure 1)

## Results

The mean and standard deviation (SD) of flexural strength for each group are summarized in Table 1. Two-way ANOVA revealed that the presence of fiber, intermediate adhesive layer, and the brand of composite material significantly affected the flexural strength (*p*< 0.05). The data were, therefore, analyzed by using post-hoc Tukey-Kramer intervals. The results showed that the mean values of flexural strength of both composites with a combination of glass fiber and intermediate adhesive layer (groups 3 and 6) were significantly higher than composites with glass fiber alone (groups 2 and 5) and the control groups (groups 1 and 4). A significant difference was also observed between the composites with glass fiber and control groups (*p*= 0.022). (Groups 1 and 2, groups 4 and 5)

**Table 1:** The mean±SD flexural strength of different groups. Different letters denote statistical difference between the groups (*p*< 0.05)

**Groups**	**Mean**	**SD**	**N**
Group 1 (Z350 Composite) Group 2 (fiber-Z350 composite) Group 3 (fiber-binding-Z350 composite)	49.61a 55.12b 68.0c	8.8746 11.1966 10.0914	10 10 10
Group 4 (P90 composite) Group 5 (fiber-P90 composite) Group 6 (fiber-binding-Z350 composite)	40.180d 51.020e 57.790f	6.6523 8.7352 10.3641	10 10 10

Filtek Z350 composite had significantly higher flexural strength than Filtek P90 composite (*p*= 0.002). The failure mode of each fiber-composite specimen is represented in Table 2. Most failure modes in were found to be mode A, except in group 5 which predominantly had failure mode B.

**Table 2:** Failure modes of fiber-composite specimens

**Groups**	**Category**			
	**A**	**B**	**C**	**D**
Group 2 (fiber-Z350 composite)	6	2	1	1
Group 3 (fiber-bond-Z350 composite)	7	2	1	0
Group 5 (fiber-P90 composite)	3	6	0	1
Group 6 (fiber-bond-P90 composite)	8	2	0	0

A: cohesive failure in composite

B: adhesive failure in composite-fiber interface

C: partial breakage of the composite and fiber

D: complete breakage of the composite and fiber

## Discussion

Currently, the interest for using FRC is rapidly increasing and this material seems to be accepted by clinicians for many clinical applications in dentistry[9, 22] In the current study, a pre-impregnated glass fiber was employed for reinforcement of composite not only because of its satisfactory esthetic aspects but also because the glass fiber can tolerate the tensile stress and prevent crack propagation in resin composite.[5-6] Moreover, this study measured the flexural strength to determine the bond reliability between the tested composites and pre-impregnated glass fiber since flexural strength test represents a viable tool to measure the bond strength.

The results of our study showed that the presence of pre-impregnated glass fiber significantly increased the flexural strength of both nanofilled and silorane-based composites. Previous studies also pointed out that the fiber reinforcement increased the mechanical properties of composite materials.[12-15,23]

Kamble *et al.* concluded that glass fiber reinforcement resulted in higher flexural strength in polymethylmethacrylate resin and bis-acryl composites.[24] Agrawal *et al.* showed that fiber inserts in class II composite restoration significantly reduced the microleakage of nanofilled and silorane-based composite.[17]

In the current study, although the glass fiber was pre-impregnated with Bis-GMA and UDMA resin, the strengthening effect of fiber was observed in silorane-based composite. It could be assumed that Filtek P90 silorane composite is compatible with methacrylate-based adhesive. This finding is in conformity with the result of Oskoee *et al.* which concluded that pre-impregnated glass fiber in conjunction with silorane composite reduced cuspal deflection of endodontically-treated maxillary premolars.[19]

However, Ghulman *et al.* revealed that the bond strength of silorane composite with different adhesives was insufficient and silorane composite should be compatible only with its specific adhesive.[25] Soldo *et al.* reported that only silorane composite and its adhesive had less microleakage in class V restorations and the combination of silorane composite with Adper Easy one (methacrylate adhesive) was not well-matched in their study.[26] Similar result was achieved by Duarte *et al.* who noticed the insufficient bond strength of methacrylate-based adhesive and silorane for dentin.[27]

These aforementioned studies have used hydrophilic adhesive in conjunction with silorane composite. Since the siloranes are highly hydrophobic, the hydrophobicity of adhesive resin is essential for adequate wetting and adhering to Filtek silorane composites, as it was seen in silorane adhesive bond. Moreover, according to the manufacturer, silorane restorations can be repaired with a conventional methacrylate composite system by using a dimethacrylate-based intermediate resin layer.[28] In our study, significant increase in flexural strength was observed in combination of Filtek P90 composite-glass fiber. The glass fiber used in our study was pre-impregnated with a pure hydrophobic methacrylate-dimethacrylate resin; therefore, adequate bond might have been created between the glass fiber and silorane composite. However, the SBS was significantly lower for this combination compared to Filtek Z350composite-glass fiber combination.

In our study, when the surfaces of FRC were covered with an intermediate adhesive resin, significant higher mean bond strength was obtained compared with other groups, either in Filtek Z350 or Filtek P90 composite resin.

Margin bond consisted of a combination of methacrylate–dimethacrylate resin. When it is used as an intermediate layer in the glass fiber before Filtek Z350 composite, it can enhance the wettability of the nanohybrid composite (group 3). Monomers can easily diffuse to linear polymer but diffusion of monomers into cross-linked polymer is difficult to achieve.[29]

Besides, Polacek *et al.* pointed out that an intermediate layer of adhesive resin on the S2-glass FRC surface must be an additional step before incremental build up with particulate-filled composite.[20-30]

Application of silorane system adhesive bond to glass fiber prior to applying Filtek P90 composite would also increase the bond strength values significantly. Giachetti *et al.* suggested that a reliable bond could be achieved between aged silorane composite and methacrylate composite by using a silorane adhesive system as an intermediate layer; although no chemical compatibility between aged silorane substrate and the methacrylate composite was observed in their study.[28] Silorane P90 adhesive is based on methacrylate chemistry and contains phosphorylated methacrylates. The reaction of phosphate groups with oxirane in silorane composite and the acrylate group with methacrylate adhesive in pre-impregnated glass fiber might be responsible for the increase of bond strength values.[31] Compatibility of silorane P90 adhesive by either the etch-and-rinse two-step adhesive (Single bond) or by the one-step self-etch dentin bonding (Easy one) was also observed in D’Alpino *et al*.’s study when replacing the P90 primer with simplified adhesives.[32]

Analysis of the failure mode in our study showed that the failure mode of fiber-composite specimen in group 6 shifted toward the cohesive failure in silorane composite when compared with predominantly adhesive failures in group 5. Although both groups had significantly higher flexural strength than group 4, it confirms the previous results that a good adhesion between silorane composite and glass fiber was created in group 6.

In our study, nano hybrid composite (without fiber) exhibited significantly higher flexural strength than silorane composite.

Several studies demonstrated a direct relation between filler volume content, size and shape on mechanical properties.[33] Flexural strength generally is reduced by increasing the filler particle size.[34] As mentioned in Table 3, the filler volume and size of Filtek Z350 may be the possible reason for our results compared to Filtek P90 silorane composite.

**Table 3:** The employed materials and their composition

**Material**	**Manufacturer**	**Material composition**	**Weight (%)**	**Filler size**
Filtek Z350 Nanocomposite (N)	3M/ESPE, St.Paul, MN, USA	Bis-GMA, Bis-EMA, UDMA, TEGDMA/ Zirconia, Silica	78.5	Nano particle (20-70nm), Nanocluster (Average:0.6µm, particle: 2-20nm)
Filtek P90 Silorane(S)	3M/ESPE, St.Paul, MN, USA	Silorane/ Quartz, yttrium fluoride	76	0.04-1.7 µm
Nanocomposite adhesive bond (Margin bond)	Colten whaledent,Germany	Bis-GMA, Bis-EMA, UDMA, TEGDMA		
Silorane system adhesive bond	3M, ESPE St.Paul, MN, USA	TEGDMA, Phosphoric acid methacryloxyhexylesters, 1,6-hexanediol dimethacrylate		
Pre-impregnated Glass fiber (FITA/RIBBON)	Angelus Industria de Produtos Odontologicos	Glass fiber: Uni, multidirectional and braided fiber glass. Impregnated resin: Bis-GMA resin, urethane dimethacrylate, barium ceramic glass, highly dispersed silicon dioxide, catalysts, and pigments. Bonding agent: silane solution in alcohol.		

Further research with regard to other mechanical properties is still required to evaluate the compatibility of fiber and silorane composite and also the longevity of this combination especially in clinical situations.

## Conclusion

Considering the limitation of this *invitro* study, it can be stated that the use of pre-impregnated glass fiber has a positive effect on flexural strength of methacrylate and silorane base composite. Additional application of an intermediate adhesive layer on FRC surface may increase the flexural strength and adhesive strength of veneering composite to glass fiber. Moreover, the flexural strength of nanohybrid composite was better than silorane base composite resin.
